# The Effects of Boundary Conditions and Friction on the Helical Buckling of Coiled Tubing in an Inclined Wellbore

**DOI:** 10.1371/journal.pone.0162741

**Published:** 2016-09-20

**Authors:** Yinchun Gong, Zhijiu Ai, Xu Sun, Biwei Fu

**Affiliations:** 1 School of Mechanical Engineering, Southwest Petroleum University, Chengdu, China; 2 Shenzhen Limited, China National Offshore Oil Corporation, Shenzhen, China; Beihang University, CHINA

## Abstract

Analytical buckling models are important for down-hole operations to ensure the structural integrity of the drill string. A literature survey shows that most published analytical buckling models do not address the effects of inclination angle, boundary conditions or friction. The objective of this paper is to study the effects of boundary conditions, friction and angular inclination on the helical buckling of coiled tubing in an inclined wellbore. In this paper, a new theoretical model is established to describe the buckling behavior of coiled tubing. The buckling equations are derived by applying the principles of virtual work and minimum potential energy. The proper solution for the post-buckling configuration is determined based on geometric and natural boundary conditions. The effects of angular inclination and boundary conditions on the helical buckling of coiled tubing are considered. Many significant conclusions are obtained from this study. When the dimensionless length of the coiled tubing is greater than 40, the effects of the boundary conditions can be ignored. The critical load required for helical buckling increases as the angle of inclination and the friction coefficient increase. The post-buckling behavior of coiled tubing in different configurations and for different axial loads is determined using the proposed analytical method. Practical examples are provided that illustrate the influence of the angular inclination on the axial force. The rate of change of the axial force decreases with increasing angular inclination. Moreover, the total axial friction also decreases with an increasing inclination angle. These results will help researchers to better understand helical buckling in coiled tubing. Using this knowledge, measures can be taken to prevent buckling in coiled tubing during down-hole operations.

## Introduction

Coiled tubing is widely used in drilling for oil or gas. The success or failure of typical down-hole operations primarily depends on whether the coiled tubing will buckle [1]. Therefore, research on buckling behavior in coiled tubing is very meaningful.

Based on certain simplifications, scholars have conducted many studies on the buckling of drill strings. However, the effects of friction, angular inclination, boundary conditions, and gravity have often been ignored. The first paper concerning the helical buckling of a drill string in a vertical well relied on the principle of minimum potential energy and was published by Lubinski [2]. Bogy and Paslay [3] studied the stability of a pipe constrained in an inclined cylinder by applying the principle of virtual work. In this way, the critical load for sinusoidal buckling was obtained. Dawson and Paslay [4] determined an approximate solution for the linear buckling of a pipe constrained in an inclined hole. Notably, the buckling behavior of a tubular string in an inclined wellbore is more complicated than that in a horizontal well. Huang and Pattillo [5] obtained an analytical solution for helical buckling without considering the effects of friction using the Rayleigh-Ritz method. Mitchell [6–8] obtained buckling solutions for extended reach wells and determined the stability criteria associated with helical buckling. Pattillo and Cheatham [9] studied the helical buckling behavior of a circular column confined in a vertical well and obtained the force-pitch relationship for axial loading. Mitchell [10] derived an analytical solution for the buckling of a circular column constrained in a horizontal wellbore. The effective boundary conditions on helical buckling were obtained while neglecting friction. Kyllingstad and He [11] researched the critical load for the helical buckling of coiled tubing constrained in a curved borehole and determined the effect of the well curvature on the critical load. Cunha and Miska [12] determined the critical load ignoring friction, gravity and torque. Liu and Gao [13] investigated the critical force for sinusoidal buckling and helical buckling without considering friction, and an approximate analytical solution was obtained. Wang et al. [14] investigated the buckling behavior model for a tube in an inclined well by applying a discrete singular convolution method. The results showed that helical buckling will occur when the axial load exceeds the critical load. McCann et al. [15] experimentally investigated the helical buckling of a horizontal rod in a pipe. The effects of gravity, torsion, and axial compression on buckling for an oil drill pipe constrained in a horizontal cylinder were experimentally studied by Wicks et al. [16]. Yinchun Chen et al. [17] investigated the axial force transfer when the coiled tubing constrained in a horizontal wellbore. The experimental results indicated that coiled tubing’s axial force transfer efficiency is reduced with the growth of annular clearance. Feng Guan et al. [18] the mechanical behavior of coiled tubing when it is in a helical buckling state. The experimental results show that the pipe deformation is advanced with the growth of the axial force. Deli Gao et al. [19] obtained the effect of residual bending. They pointed out that the residual bending of coiled tubing makes it easier to take helical buckling. J.T. Miller et al. [20–21] researched the effect of friction on the helical buckling of coiled tubing through the numerical simulations and experiments. Wenjun Hang et al. [22] derived a new buckling equation of the tubular string when the friction is neglected. The article focuses on the influence of the boundary conditions on the helical buckling. These findings have been widely used in engineering practice. However, most of these models ignore the effects of inclination angle, boundary conditions, and friction, among other factors. In an inclined wellbore, coiled tubing typically undergoes first sinusoidal buckling and then helical buckling, and friction, inclination angle, and gravity are all important factors affecting the critical buckling load.

The influences of boundary conditions, friction, and inclination angle are discussed in this work. The energy method is used to obtain various critical loads by assuming different buckling configurations. Firstly, the equations for the buckling of coiled tubing in an inclined wellbore under an axial load are developed. Secondly, an approximate analytical solution for the static buckling problem is obtained using the perturbation method. Finally, a detailed analysis of the effects of friction and inclination angle on the critical load for helical buckling is performed.

## Theoretical Model

### Assumptions

**Fig 1:**
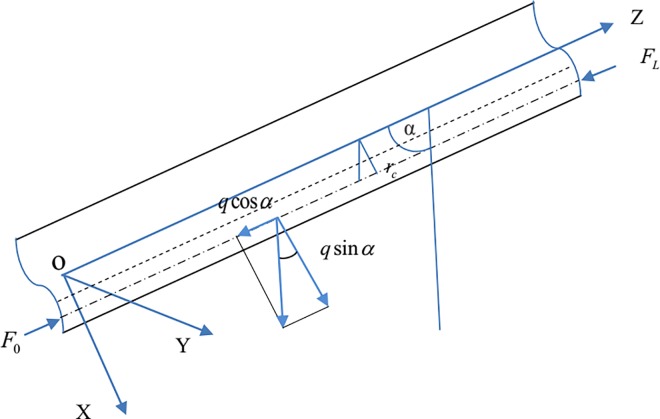
Coiled tubing in an inclined wellbore (side view).

1. We assume that the inner diameter and inclination angle of the wellbore are constants.
2. We assume that the coiled tubing and wellbore are round and maintain continuous contact.
3. We ignore the effects of torque and the heat generated by friction.
4. The clearance (*r*_*c*_, see Fig 1) between the axis of the coiled tubing and the borehole axis is assumed to be small.
5. The coiled tubing is assumed to remain within the elastic deformation regime.

## Geometry and mechanical analysis

Fig 1 shows the coordinate system. At a point *O*′ on the Z axis, the angular, radial linear, and axial linear displacements can be expressed as *θ*(*z*), r(*z*), and u(*z*), respectively. *α* is used to indicate the angle between the vertical line and the Z axis. *u* represents the axial displacement from the load end to the bottom end of the coiled tubing. The vector ${\text{r}}_{o^{\prime }}\left(z\right)$ represents the spatial position of the coiled tubing’s axis.

$${\text{r}}_{o^{\prime }}\left(z\right)=r\cos\theta \vec{i}+r\sin\theta \vec{j}+\left(z-u\right)\vec{k}.$$ (1)

The forces acting on the coiled tubing include the compressive force *F*_*L*_, the normal contact force *N*, the friction force *f*, and the weight *q* of the coiled tubing and the fluid contained therein. We assume that the axial displacement of the coiled tubing at *z* = 0 is zero. u_*a*_(*z*) represents the displacement induced by the axial force. u_*b*_(*z*) represents the displacement caused by buckling or lateral bending. Therefore, the total axial displacement u(*z*) is

$$\text{u}\left(z\right){=\text{u}}_{a}\left(z\right)+{\text{u}}_{b}\left(z\right)=\frac{1}{EA}\int_{0}^{z}F\left(z\right)dz+\frac{1}{2}\int_{0}^{z}\left[{\left(\frac{dr}{dz}\right)}^{2}+{\left(r\frac{d\theta }{dz}\right)}^{2}\right]dz,$$ (2)

where $A=\pi \left(R_{P}^{2}-r_{P}^{2}\right)$ is the cross-sectional area of the pipe in *m*^2^.

**Fig 2:**
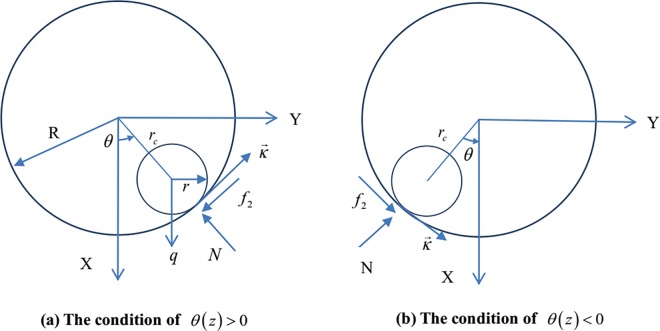
Angular displacement of the coiled tubing.

Fig 2 shows the angular displacement (*θ*(*z*)) of the pipe. For a coiled tubing and wellbore in continuous contact, *r*_*c*_ represents the distance between the axial line of the coiled tubing and the Z axis. *f*_1_(*z*) is the axial component of the sliding friction coefficient, whereas *f*_2_(*z*) is the lateral component. The directions of the lateral friction force $-f_{2}N\vec{\kappa }$ and $\vec{\kappa }$ are opposite when the coiled tubing slides upward toward the right-hand side (*θ*(*z*) > 0), as shown in Fig 2. By contrast, the directions of the lateral friction force and $\vec{\kappa }$ are the same when the coiled tubing is sliding upward toward the left-hand side (*θ*(*z*) < 0)(again, see Fig 2).

### Buckling equations for coiled tubing and their normalization

This paper consider the elastic deformation energy (*U*) and the total work (*W*) (see Appendix A in S1 File). The total energy (П) of the system is the difference between the total work and the elastic potential energy. For *r* < *r*_*c*_, there is no contact between the coiled tubing and the borehole wall. We assume that the tubing and wall are in continuous contact. Therefore, the value of *r* is a constant, *r*_*c*_, and *N*(*z*) > 0. Thus, the total energy is

$$\begin{array}{l} \prod =U-W=\frac{EIr_{\text{c}}^{2}}{2}\int_{0}^{L}\left[{\left(\frac{d^{2}\theta }{dz^{2}}\right)}^{2}+{\left(\frac{d\theta }{dz}\right)}^{4}\right]dz-\int_{0}^{u_{b}(L)}F_{L}du_{b}(L)+\int_{0}^{L}\int_{0}^{u_{b}(z)}f_{1}\left(z\right)N\left(z\right)du_{a}(z)dz\\ \;-\int_{0}^{L}\int_{0}^{u_{b}(z)}q\cos\alpha du_{a}(z)dz+q\sin\alpha r_{c}\int_{0}^{L}\left(1-\cos\theta \right)dz+\int_{0}^{L}sign\left(\theta \right)\int_{0}^{\theta \left(z\right)}f_{2}Nr_{c}d\varphi dz \end{array}.$$ (3)

When the buckled coiled tubing changes to a new equilibrium configuration, the net work is converted into elastic potential energy. Therefore, we use the concept of virtual work to determine *δ*∏ = 0. Considering the effects of the inclination angle and friction, the buckling equations are derived as shown in Appendix A in S1 File.

The buckling equation for coiled tubing in an inclined wellbore can be expressed as

$$EIr_{c}^{2}\frac{d^{4}\theta }{dz^{4}}-6EIr_{c}^{2}{\left(\frac{d\theta }{dz}\right)}^{2}\frac{d^{2}\theta }{dz^{2}}+r_{c}^{2}\frac{d}{dz}\left(F\frac{d\theta }{dz}\right)+q\sin\alpha r_{c}\sin\theta +f_{2}Nr_{c}sign\left(\theta \right)=0.$$ (4)

The first two terms in Eq 4 represent the elastic potential energy. The third term represents the work done by the axial force. The fourth term arises from the effect of gravity. Finally, the last term represents the work done by friction.

The normal contact force is

$$N=EIr_{c}\left[3{\left(\frac{d^{2}\theta }{dz^{2}}\right)}^{2}+4\frac{d\theta }{dz}\frac{d^{3}\theta }{dz^{3}}-{\left(\frac{d\theta }{dz}\right)}^{4}\right]+q\sin\alpha \cos\theta +Fr_{c}{\left(\frac{d\theta }{dz}\right)}^{2}.$$ (5)

The first term in Eq 5 represents the elastic force. The second term is the gravity component. The last term represents the effect of the axial force.

The axial force can be expressed as

$$\frac{dF\left(z\right)}{dz}=f_{1}\left(z\right)N\left(z\right)-q\cos\alpha .$$ (6)

Therefore, the axial force is the interaction between the components of gravity and friction.

Given the proper boundary conditions, the normal contact force *N*(*z*), the angular displacement *θ*(*z*), and the axial force *F*(*z*) can be determined by solving Eqs 4–6. When the friction is zero and *α* = 90°, Eqs 4 and 5 are identical to the results derived by R. F. Mitchell [6]. Because these forces remain unchanged for virtual displacements, Eq 3 can be simplified to

$$\prod =\frac{EIr_{c}^{2}}{2}\int_{0}^{L}\left[{\left(\frac{d^{2}\theta }{dz^{2}}\right)}^{2}+{\left(\frac{d\theta }{dz}\right)}^{4}\right]dz-\frac{r_{c}^{2}}{2}\int_{0}^{L}F\left(z\right){\left(\frac{d\theta }{dz}\right)}^{2}dz+r_{c}\int_{0}^{L}sign\left(\theta \right)\int_{0}^{\theta \left(z\right)}f_{2}Nd\varphi dz-r_{c}q\sin\alpha \int_{0}^{L}\left(\cos\theta -1\right)dz.$$ (7)

By introducing the dimensionless total energy $\Omega =\frac{\prod }{r_{c}q\sin\alpha L}$, the dimensionless axial load $m=\frac{q\sin\alpha }{EIr_{c}\mu^{4}}$, the dimensionless distance *ς* = *μz*, the dimensionless normal contact force $n=\frac{N}{EIr_{c}\mu^{4}}$, and the parameter $\mu =\sqrt{\frac{F}{2EI}}$, Eqs 4–7 can be rewritten as

$$\frac{d^{4}\theta }{d\varsigma^{4}}-6{\left(\frac{d\theta }{d\varsigma }\right)}^{2}\frac{d^{2}\theta }{d\varsigma^{2}}+2\frac{d^{2}\theta }{d\varsigma^{2}}+m\sin\theta +f_{2}nsign\left(\theta \right)=0,$$ (8)

$$n=3{\left(\frac{d^{2}\theta }{d\varsigma^{2}}\right)}^{2}+4\frac{d\theta }{d\varsigma }\frac{d^{3}\theta }{d\varsigma^{3}}-{\left(\frac{d\theta }{d\varsigma }\right)}^{4}+m\cos\theta +2{\left(\frac{d\theta }{d\varsigma }\right)}^{2},$$ (9)

$$\frac{1}{m}\frac{dm}{d\varsigma }=\mu f_{1}nr_{c}-\mu m\cot\alpha ,$$ (10)

$$\Omega =\frac{1}{\varsigma_{L}}\frac{1}{2m}\int_{0}^{\varsigma_{L}}\left[{\left(\frac{d^{2}\theta }{d\varsigma^{2}}\right)}^{2}+{\left(\frac{d\theta }{d\varsigma }\right)}^{4}-2{\left(\frac{d\theta }{d\varsigma }\right)}^{2}\right]d\varsigma +\frac{1}{\varsigma_{L}}\frac{1}{m}\int_{0}^{\varsigma_{L}}sign\left(\theta \right)\int_{0}^{\theta \left(\varsigma \right)}f_{2}nd\vartheta d\varsigma +\frac{1}{\varsigma_{L}}\int_{0}^{\varsigma_{L}}\left(1-\cos\theta \right)d\varsigma .$$ (11)

## Boundary Condition Analysis

### Natural boundary conditions

For a pinned end at *z* = z*, the boundary conditions are

$$\theta (z^{*})=0,\;\delta \theta (z^{*})=0,{\left(\frac{d^{2}\theta }{dz^{2}}\right)}_{z^{*}}=0,\;\delta {\left(\frac{d^{2}\theta }{dz^{2}}\right)}_{z^{*}}=0.$$ (12)

For a fixed end at *z* = z*, the boundary conditions are

$$\theta (z^{*})=0,\;\delta \theta (z^{*})=0,{\left(\frac{d\theta }{dz}\right)}_{z^{*}}=0,\;\delta {\left(\frac{d\theta }{dz}\right)}_{z^{*}}=0.$$ (13)

Conditions corresponding to a frictionless, massless pipe that is freed at one end (*z* = *L*) and pinned at the other end are considered and are expressed as follows:

$$\theta (z)=0,{\left(\frac{d^{2}\theta }{dz^{2}}\right)}_{z=0}=0,{\left(\frac{d^{2}\theta }{dz^{2}}\right)}_{z=L}=0,\;\mathrm{and}\;{\left(\frac{d^{3}\theta }{dz^{3}}\right)}_{z=L}=0.$$ (14)

Thus the buckling equation (Eq 4) for coiled tubing in an inclined wellbore can be simplified to

$$\frac{d^{4}\theta }{dz^{4}}-6{\left(\frac{d\theta }{dz}\right)}^{2}\frac{d^{2}\theta }{dz^{2}}+\frac{F}{EI}\frac{d^{2}\theta }{dz^{2}}=0.$$ (15)

We assume that *θ* = *υz* satisfies both the boundary conditions (Eq 14) and the buckling equation (Eq 15) for *υ* equal to any real number. The solution $\upsilon =\sqrt{\frac{F}{2EI}}$ representing the helical buckling configuration for a piece of coiled tubing was obtained by A. Lubinski in 1962 by applying the principle of minimum potential energy. However, for the given boundary conditions, we cannot arrive at the same solution. This means that the definitions of the boundary conditions that were previously used are not appropriate for the problem considered in this paper. We must further study this problem to obtain the correct solution. In this case, *θ* = *υz* satisfies the boundary conditions for the free end. However, this solution does not satisfy the other boundary conditions. For instance, it does not correspond to the conditions for fixed and pinned ends. Therefore, at the loading end (z* = *L*), we arrive at *θ*(*L*) = *υL* ≠ 0 and ${\left(\frac{d\theta }{dz}\right)}_{z=L}=\upsilon \neq 0$.

In this paper, the axial load work and the elastic deformation energy are given as shown in Appendix B in S1 File. Substituting Eqs (A-10), (A-13), (B-2), and (B-4) into $\delta \prod =\delta U_{b}-\delta W_{Fz,b}-\delta W_{G_{2}}-\delta W_{f_{2}}=0$ yields

$$\begin{array}{l} \delta \prod =\int_{0}^{L}\left[\frac{d^{4}\theta }{dz^{4}}-6\frac{d^{2}\theta }{dz^{2}}{\left(\frac{d\theta }{dz}\right)}^{2}+\frac{d}{dz}\left(\frac{F}{EI}\frac{d\theta }{dz}\right)+\frac{q\sin\alpha }{EIr_{c}}\sin\theta +\frac{f_{2}Nsign\left(\theta \right)}{EIr_{c}}\right]\delta \theta dz\\ \;+{\left[\frac{d^{2}\theta }{dz^{2}}\delta \left(\frac{d\theta }{dz}\right)\right]}_{0}^{L}-{\left[\left(\frac{d^{3}\theta }{dz^{3}}+\frac{F}{EI}\frac{d\theta }{dz}-2{\left(\frac{d\theta }{dz}\right)}^{3}\right)\delta \theta \right]}_{0}^{L}=0 \end{array}.$$ (16)

Because *δθ* is arbitrary, *δ*∏ = 0 requires that the buckling equation for the coiled tubing and the natural boundary conditions must satisfy the following relationships:

$$\frac{d^{4}\theta }{dz^{4}}-6\frac{d^{2}\theta }{dz^{2}}{\left(\frac{d\theta }{dz}\right)}^{2}+\frac{d}{dz}\left(\frac{F}{EI}\frac{d\theta }{dz}\right)+\frac{q\sin\alpha }{EIr_{c}}\sin\theta +\frac{f_{2}Nsign\left(\theta \right)}{EIr_{c}}=0,$$ (17)

$${\left[\frac{d^{2}\theta }{dz^{2}}\delta \left(\frac{d\theta }{dz}\right)\right]}_{0}^{L}-{\left[\left(\frac{d^{3}\theta }{dz^{3}}-2{\left(\frac{d\theta }{dz}\right)}^{3}+\frac{F}{EI}\frac{d\theta }{dz}\right)\delta \theta \right]}_{0}^{L}=0.$$ (18)

After nondimensionalization, Eq 17 becomes Eq 8, whereas Eq 18 becomes

$${\left[\frac{d^{2}\theta }{d\varsigma^{2}}\delta \left(\frac{d\theta }{d\varsigma }\right)\right]}_{0}^{\varsigma_{L}}-{\left[\left(\frac{d^{3}\theta }{d\varsigma^{3}}-2{\left(\frac{d\theta }{d\varsigma }\right)}^{3}+2\frac{d\theta }{d\varsigma }\right)\delta \theta \right]}_{0}^{\varsigma_{L}}=0.$$ (19)

Without considering the effect of friction, Miska analyzed the boundary conditions for a bottom hole assembly in 1986. However, the natural boundary conditions for this problem were not investigated. The natural boundary conditions can be expressed as

$${\left[\frac{d^{2}\theta }{d\varsigma^{2}}\right]}_{\varsigma =\varsigma^{*}}=0,\;\text{or}\;{\left[\delta \left(\frac{d\theta }{d\varsigma }\right)\right]}_{\varsigma =\varsigma^{*}}=0,$$ (20)

$${\left[\frac{d^{3}\theta }{d\varsigma^{3}}-2{\left(\frac{d\;\theta }{d\varsigma }\right)}^{3}+2\frac{d\;\theta }{d\varsigma }\right]}_{\varsigma =\varsigma^{*}}=0\;,\;or\;{\left[\delta \theta \right]}_{\varsigma =\varsigma^{*}}=0.$$ (21)

For instance, ${\left[\frac{d^{2}\theta }{d\varsigma^{2}}\right]}_{\varsigma =\varsigma^{*}}=0$ and ${\left[\delta \theta \right]}_{\varsigma =\varsigma^{*}}=0$ are the boundary conditions for a pinned end, whereas the equations ${\left[\delta \theta \right]}_{\varsigma =\varsigma^{*}}=0$ and ${\left[\delta \left(\frac{d\theta }{d\varsigma }\right)\right]}_{\varsigma =\varsigma^{*}}=0$ are the boundary conditions for a fixed end. The equations ${\left[\frac{d^{2}\theta }{d\varsigma^{2}}\right]}_{\varsigma =\varsigma^{*}}=0$ and ${\left[\frac{d^{3}\theta }{d\varsigma^{3}}-2{\left(\frac{d\theta }{d\varsigma }\right)}^{3}+2\frac{d\theta }{d\varsigma }\right]}_{\varsigma =\varsigma^{*}}=0$ are the boundary conditions of the free end. The natural boundary condition can apply to a fixed or a pinned end. At the same time, the natural condition (Eq 19) must satisfy the solution for the buckling equation (Eq 17).

Now, let us analyze the boundary conditions for weightless coiled tubing. Because $\frac{d^{2}\theta }{dz^{2}}=\frac{d^{3}\theta }{dz^{3}}=0$ for any real constants *υ* and *z*, ${\left[\frac{d^{2}\theta }{dz^{2}}\delta \left(\frac{d\theta }{dz}\right)\right]}_{0}^{L}={\left[\frac{d^{3}\theta }{dz^{3}}\delta \theta \right]}_{0}^{L}=0$. Notably, *δθ* = *Lδυ* ≠ 0 at *z* = *L* and *δθ* = *zδυ* = 0 at *z* = 0. Therefore, $\upsilon \left(\upsilon^{2}-\frac{F}{2EI}\right)L\delta \upsilon =0$ can be determined using Eq 18 because *δυ* is not equal to zero. Thus, the following conclusions are obtained: *υ* = 0 is a trivial solution. It represents the coiled tubing without any buckling. Meanwhile, $\upsilon =\frac{d\theta }{dz}=\pm \sqrt{\frac{F}{2EI}}$ is an also valid, non-trivial solution. This is the same conclusion obtained by Lubinski et al. in 1962.

### Critical loads for helical buckling with different boundary conditions

For boundary conditions corresponding to two pinned ends, the total dimensionless energy for a length of helically buckled coiled tubing constrained in an inclined wellbore at the onset of helical buckling is derived as follows (see Appendix C in S1 File):

$$\Omega_{h}=\left(\frac{1}{2m}-\frac{\pi f_{2}}{10m}\right)p_{h}^{4}+\left(\frac{\pi f_{2}}{3m}-\frac{1}{m}\right)p_{h}^{2}+\frac{2f_{2}}{\pi }+1,$$ (22)

where *p*_*h*_ is the angular frequency of the angular displacement.

As helical buckling begins, we can arrive at the following conclusions based on the law of energy conservation. Part of the work is converted into heat energy by friction. Because the coiled tubing is raised, part of the energy is also converted into gravitational potential energy. The rest of the work is converted into elastic deformation energy. Thus, the total energy satisfies the relationship $\Pi =U_{b}-W_{f}-W_{G_{2}}-W_{Fb}=0$, i.e., Ω_*h*_ = 0. Therefore,

$$m==\frac{\left[\left(\frac{\pi f_{2}}{10}-\frac{1}{2}\right)p_{h}^{4}+\left(1-\frac{\pi f_{2}}{3}\right)p_{h}^{2}\right]\pi }{2f_{2}+\pi }.$$ (23)

Given a helically buckled coiled tubing, the maximum value of *m* is the critical load. The critical value of *p*_*h*_ can be obtained by considering the beginning of helical buckling. Substituting $\frac{dm}{dp_{h}}=0$ into Eq 23 yields

$$p_{h,crh}=\sqrt{\frac{5\pi f_{2}-15}{3\pi f_{2}-15}}.$$ (24)

#### Boundary conditions corresponding to two pinned ends

We assume that the coiled tubing is slowly sliding. The integer *k* is used to represent the number of helical buckling points for a section of coiled tubing of length *ς*_*L*_. For the case in which both ends are pinned, we can determine that *θ*(*ς*_*L*_) = *p*_*h*_*ς*_*L*_ = 2*kπ*. Substituting $p_{h}=\frac{2k\pi }{\varsigma_{L}}$ into both Eqs 23 and 24 yields

$$m=\frac{\left[\left(\frac{\pi f_{2}}{10}-\frac{1}{2}\right){\left(\frac{2k\pi }{\varsigma_{L}}\right)}^{4}+\left(1-\frac{\pi f_{2}}{3}\right){\left(\frac{2k\pi }{\varsigma_{L}}\right)}^{2}\right]\pi }{2f_{2}+\pi },$$ (25)

$$k_{crh}=\max\left\{1,\mathrm{int}\left[\sqrt{\frac{5\pi f_{2}-15}{3\pi f_{2}-15}}\frac{\varsigma_{L}}{2\pi }+0.5\right]\right\}.$$ (26)

For a given friction coefficient and dimensionless length, the critical value *k*_*crh*_ can be calculated using Eq 26. The dimensionless critical load *m*_*crh*_ for helical buckling can be obtained by substituting Eq 26 into Eq 25. As *ς*_*L*_ → ∞, $p_{h}=\frac{2k\pi }{\varsigma_{L}}$ approaches $p_{h,crh}=\sqrt{\frac{5\pi f_{2}-15}{3\pi f_{2}-15}}$. When *ς*_*L*_ → ∞, the value of *m*_*crh*_ approaches

$$m_{crh}=\frac{5\pi^{3}f_{2}^{2}-30\pi^{2}f_{2}+45\pi }{-36\pi f_{2}^{2}+\left(180-18\pi^{2}\right)f_{2}+90\pi }.$$ (27)

#### Boundary conditions corresponding to a free end and a pinned end

In this section, we consider the boundary conditions corresponding to a length of coiled tubing with a free end (*ς* = *ς*_*L*_) and a pinned end (*ς* = 0). It can be assumed that the form of the helix is *θ*(*ς*) = *p*_*h*_*ς*. Substituting *θ*(*ς*) = *p*_*h*_*ς* into Eq 19 yields *p*_*h*_ = 1. Then, substituting *p*_*h*_ = 1 into Eq 22 yields

$$\Omega_{h}=\frac{7\pi f_{2}}{30m}-\frac{1}{2m}+\frac{2f_{2}}{\pi }+1.$$ (28)

Thus, we can apply the law of conservation of energy (Ω_*h*_ = 0) to determine the dimensionless critical buckling load for helical buckling from Eq 28.

$$m_{crh}=\frac{15\pi -7\pi^{2}f_{2}}{60f_{2}+30\pi }.$$ (29)

## Frictional Analysis and Axial Load Transfer

A length of coiled tubing constrained in an inclined wellbore can assume three types of equilibrium states: helical, sinusoidal, and straight-lined. Because of the influence of the inclination angle and axial friction, buckling behavior will initially occur near either the bottom or loading end. The angle of inclination can be divided into two regimes based on the self-locking angle due to friction. The maximum axial force will occur near the loading end when the angle of inclination is greater than the self-locking angle, whereas the maximum axial force will appear at the bottom end when the angle of inclination is less than the self-locking angle.

### The first case of inclination angle

Only a portion of the coiled tubing will form a sinusoidal shape near the loading end when $\arctan\frac{1}{f}\leq \alpha$. Therefore, the next section addresses the case in which $\arctan\frac{1}{f}\leq \alpha$.

#### The first case of compressive force

When $0<F_{L}<F_{crs}=2\sqrt{1+4.348f_{2}^{2/3}}\sqrt{q\sin\alpha EI/r_{c}}$, the coiled tubing retains a straight shape. In this case, *θ*(*z*) = 0 is a stable solution. Because the coiled tubing does not undergo sinusoidal buckling, the contact force *N* remains constant. When *F*_*L*_ is applied at the loading end (z* = *L*), the axial force on the coiled tubing at any location can be expressed as

$$F(z)=\max\left\{0,F_{L}+(q\cos\alpha -fq\sin\alpha )(z_{L}-z)\right\}.$$ (30)

In the case of *F*_*L*_ ≤ (*fq* sin *α* − *q* cos *α*)*z*_*L*_, the axial force is zero in the section of the coiled tubing where $0<z\leq z^{*}=z_{L}-\frac{F_{L}}{fq\sin\alpha -q\cos\alpha }$ because of friction. Only when *F*_*L*_ > (*fq* sin *α* − *q* cos *α*)*z*_*L*_ can the axial force be transmitted to the bottom of the coiled tubing (z* = 0). Therefore, the axial load at the dead end can be expressed as

$$F(0)=\max\left\{0,F_{L}+(q\cos\alpha -fq\sin\alpha )z_{L}\right\}.$$ (31)

#### The second case of compressive force

When *F*_*crs*_ ≤ *F*_*L*_ < *F*_*crh*_, the periodic solution is a stable solution (see Appendix D in S1 File) and can be expressed as

$$\theta (\varsigma ,t)=\sqrt{\frac{2\left(1-m\right)}{3}}\sin\varsigma .$$ (32)

It is worth noting that the axial force on the coiled tubing may be a function of time. Substituting Eq 32 into Eq 9 yields

$$n=\frac{1}{6}+\frac{5\text{m}}{6}-\left(\frac{17}{9}-\frac{41m}{18}+\frac{7m^{2}}{18}\right)\cos2\varsigma -\frac{1+m^{2}-2m}{18}\cos\left(4\varsigma \right).$$ (33)

Neglecting the periodic terms, the equation for the dimensionless contact force can be simplified to

$$n=\frac{1}{6}+\frac{5\text{m}}{6}.$$ (34)

Therefore, the axial force on the coiled tubing at any location can be described by

$$N=\frac{r_{c}F^{2}}{24EI}+\frac{5q\sin\alpha }{6},$$ (35)

$$\frac{dF\left(z\right)}{dz}=\frac{fr_{c}}{24EI}F^{2}+\frac{5fq\sin\alpha }{6}-q\cos\alpha .$$ (36)

By substituting Eq 35 into Eq 36, the solution for *F*(*z*) can be expressed as follows.

For the case of $\frac{5}{6}f\geq \cot\alpha$,

$$F_{s}(z)=2\sqrt{\frac{EIq\left(5f\sin\alpha -6\cos\alpha \right)}{fr_{c}}}\tan\left[\frac{z-z_{L}}{12}\sqrt{\frac{fr_{c}q\left(5f\sin\alpha -6\cos\alpha \right)}{EI}}+\arctan\left(\frac{F_{L}}{2}\sqrt{\frac{fr_{c}}{EIq\left(5f\sin\alpha -6\cos\alpha \right)}}\right)\right].$$ (37)

For the case of $\frac{5}{6}f<\cot\alpha$,

$$F_{s}(z)=2\sqrt{\frac{EIq\left(6\cos\alpha -5f\sin\alpha \right)}{fr_{c}}}\tanh\left[\frac{z_{L}—z}{12}\sqrt{\frac{fr_{c}q\left(6\cos\alpha -5f\sin\alpha \right)}{EI}}+\mathrm{arc}\;\tanh\left(\frac{F_{L}}{2}\sqrt{\frac{fr_{c}}{EIq\left(6\cos\alpha -5f\sin\alpha \right)}}\right)\right].$$ (38)

In the case of *F*(0) < *F*_*crs*_, only a portion of the coiled tubing (*z*_*crs*_ ≤ *z* ≤ *L*) will exhibit a sinusoidal buckling shape near the loading end, whereas the remainder of the coiled tubing (0 ≤ *z* ≤ *z*_*crs*_) will retain a straight shape near the bottom of the wellbore. The point at which sinusoidal buckling is induced (*z*_*crs*_) in the coiled tubing can be determined by solving Eq 37 or 38. The axial load on the coiled tubing in the straight-line state can be calculated using Eq 39. Thus, the axial load *F*(0) at the dead end can be obtained from Eq 40.

$$F(z)=\max\left\{0,F_{crs}+(q\cos\alpha -fq\sin\alpha )(z_{crs}-z)\right\},$$ (39)

$$F(0)=\max\left\{0,F_{crs}+(q\cos\alpha -fq\sin\alpha )z_{crs}\right\}.$$ (40)

#### The third case of compressive force

When *F*_*L*_ ≥ *F*_*crh*_, part of the coiled tubing is in a helical buckling state. The helical solution is a stable solution (see Appendix E in S1 File).

θ(ς)=ς. (41)

Substituting Eq 41 into Eq 9 yields

n=1+mcosς. (42)

Compared to the linear term, the periodic term is very small. Therefore, we can ignore the periodic term when calculating the axial force. Substituting both Eq 42 and $n=\frac{N}{EIr_{c}\mu^{4}}$ into Eq 6 yields

$$\frac{dF\left(z\right)}{dz}=\frac{fr_{c}}{4EI}F^{2}-q\cos\alpha .$$ (43)

When *F*_*L*_ is applied at the loading end, the axial force on the coiled tubing can be expressed as

$$F_{h}\left(z\right)=2\sqrt{\frac{EIq\cos\alpha }{fr_{c}}}\tanh\left[\frac{z_{L}-z}{2}\sqrt{\frac{fr_{c}q\cos\alpha }{EI}}+\mathrm{arc}\;\tanh\left(\frac{F_{L}}{2}\sqrt{\frac{fr_{c}}{EIq\cos\alpha }}\right)\right].$$ (44)

When *F*_*h*_(0) > *F*_*crh*_, the entirety of the coiled tubing is in a helical buckling state. When *F*_*h*_(0) < *F*_*crh*_, only part of the coiled tubing near the loading end (*z*_*crh*_ < *z* ≤ z_*L*_) is in a helical buckling state, whereas the section toward the bottom of the coiled tubing (0 ≤ *z* ≤ *z*_*crs*_) will be straight or sinusoidal. The point at which sinusoidal buckling is induced (*z*_*crs*_) in the coiled tubing can be determined by solving Eq 37 or 38. The critical point for helical buckling (*z*_*crh*_) can be calculated using Eq 44, as follows:

$$z_{crh}=z_{L}-2\sqrt{\frac{EI}{fr_{c}q\cos\alpha }}\left[\mathrm{arc}\;\tanh\left(\frac{F_{crh}}{2}\sqrt{\frac{fr_{c}}{EIq\cos\alpha }}\right)-\mathrm{arc}\;\tanh\left(\frac{F_{L}}{2}\sqrt{\frac{fr_{c}}{EIq\cos\alpha }}\right)\right].$$ (45)

In the case of *F*_*h*_(0) < *F*_*crh*_, only part of the pipe is in a helical buckling state. Therefore, we first calculate $F_{s}^{*}(z)$.

For the case of $\frac{5}{6}f\geq \cot\alpha$,

$$F_{s}^{*}(z)=2\sqrt{\frac{EIq\left(5f\sin\alpha -6\cos\alpha \right)}{fr_{c}}}\tan\left[\frac{z-z_{crh}}{12}\sqrt{\frac{fr_{c}q\left(5f\sin\alpha -6\cos\alpha \right)}{EI}}+\arctan\left(\frac{F_{crh}}{2}\sqrt{\frac{fr_{c}}{EIq\left(5f\sin\alpha -6\cos\alpha \right)}}\right)\right].$$ (46)

For the case of $\frac{5}{6}f<\cot\alpha$,

$$F_{s}^{*}(z)=2\sqrt{\frac{EIq\left(6\cos\alpha -5f\sin\alpha \right)}{fr_{c}}}\tanh\left[\frac{z_{crh}—z}{12}\sqrt{\frac{fr_{c}q\left(6\cos\alpha -5f\sin\alpha \right)}{EI}}+\mathrm{arc}\;\tanh\left(\frac{F_{crh}}{2}\sqrt{\frac{fr_{c}}{EIq\left(6\cos\alpha -5f\sin\alpha \right)}}\right)\right].$$ (47)

If $F_{s}^{*}(0)>F_{crs}$, this indicates that the remainder of the coiled tubing experiences sinusoidal buckling and $F(0)=F_{s}^{*}(0)$. Otherwise, we must calculate $z_{crs}^{*}$ using Eq 48 or 49.

When $\frac{5}{6}f\geq \cot\alpha$,

$$F_{crs}=2\sqrt{\frac{EIq\left(5f\sin\alpha -6\cos\alpha \right)}{fr_{c}}}tan\left[\frac{z_{crs}^{*}-z_{crh}}{12}\sqrt{\frac{fr_{c}q\left(5f\sin\alpha -6\cos\alpha \right)}{EI}}+\arctan\left(\frac{F_{crh}}{2}\sqrt{\frac{fr_{c}}{EIq\left(5f\sin\alpha -6\cos\alpha \right)}}\right)\right].$$ (48)

When $\frac{5}{6}f<\cot\alpha$,

$$F_{crs}=2\sqrt{\frac{EIq\left(6\cos\alpha -5f\sin\alpha \right)}{fr_{c}}}\tanh\left[\frac{z_{crh}—z_{crs}^{*}}{12}\sqrt{\frac{fr_{c}q\left(6\cos\alpha -5f\sin\alpha \right)}{EI}}+\mathrm{arc}\;\tanh\left(\frac{F_{crh}}{2}\sqrt{\frac{fr_{c}}{EIq\left(6\cos\alpha -5f\sin\alpha \right)}}\right)\right].$$ (49)

The coiled tubing is in a sinusoidal buckling state over the interval $\left(z_{crs}^{*},z_{crh}\right)$. Therefore, the axial load on the coiled tubing over the interval $\left(z_{crs}^{*},z_{crh}\right)$ can be determined by solving Eq 46 or 47. Meanwhile, the coiled tubing remains straight over the interval $\left(0,z_{crs}^{*}\right)$, and the axial load on the coiled tubing over this interval can therefore be determined by solving Eq 50. In this case, the axial force at the dead end can be determined using Eq 51.

$$F(z)=\max\left\{0,F_{crs}+(q\cos\alpha -fq\sin\alpha )(z_{crs}^{*}-z)\right\},$$ (50)

$$F(0)=\max\left\{0,F_{crs}+(q\cos\alpha -fq\sin\alpha )z_{crs}^{*}\right\}.$$ (51)

The total axial friction can be expressed as

$$\Delta F=F_{L}-F(0).$$ (52)

### The second case of inclination angle

#### The first case of compressive force

Now, let us analyze the case of $\alpha <\arctan\frac{1}{f}$. When 0 < *F*_*L*_ < *F*_*crs*_ − (*q* cos *α* − *fq* sin *α*)*z*_*L*_, the coiled tubing takes on a straight shape and *θ*(*z*) = 0 is a stable solution. Therefore, the contact force between the pipe and wellbore remains at a constant value. When *F*_*L*_ is applied at the loading end, the axial force over the interval (0 < *z* ≤ *z*_*L*_) is

$$F(z)=F_{L}+(q\cos\alpha -fq\sin\alpha )(z_{L}-z).$$ (53)

In this situation, the total dissipated axial force is a constant.

$$\Delta F=F(0)-F_{L}=(q\cos\alpha -fq\sin\alpha )z_{L}.$$ (54)

#### The second case of compressive force

When *F*_*crs*_ − (*q* cos *α* − *fq* sin *α*)*z*_*L*_ ≤ *F*_*L*_ < *F*_*crs*_ − (*q* cos *α* − *fq* sin *α*)(*z*_*L*_ − *z*_max_), only part of the coiled tubing $\left(0<z\leq z_{crs}^{0}\right)$ assumes a sinusoidal buckling shape near the bottom end. The parameter *z*_max_ represents the maximum length when the coiled tubing is in buckling state. The remainder of the coiled tubing retains a straight shape. The points *z*_max_ and $z_{crs}^{0}$ can be calculated using Eqs 55 and 56, respectively:

$$F_{crh}=2\sqrt{\frac{EIq\left(6\cos\alpha -5f\sin\alpha \right)}{fr_{c}}}\tanh\left[\frac{z_{\max}}{12}\sqrt{\frac{fr_{c}q\left(6\cos\alpha -5f\sin\alpha \right)}{EI}}+\mathrm{arc}\;\tanh\left(\frac{F_{crs}}{2}\sqrt{\frac{fr_{c}}{EIq\left(6\cos\alpha -5f\sin\alpha \right)}}\right)\right],$$ (55)

and

$$z_{crs}^{0}=z_{L}-\frac{F_{crs}-F_{L}}{q\cos\alpha -fq\sin\alpha },$$ (56)

where $z_{crs}^{0}$ is the position of buckling-induced.

Over the interval $\left(0,z_{crs}^{0}\right)$, the coiled tubing takes on a sinusoidal shape, which can be determined from the axial force along the tubing.

$$F_{crs}^{\circ }(z)=2\sqrt{\frac{EIq\left(6\cos\alpha -5f\sin\alpha \right)}{fr_{c}}}\tanh\left[\frac{z_{crs}^{0}—z}{12}\sqrt{\frac{fr_{c}q\left(6\cos\alpha -5f\sin\alpha \right)}{EI}}+\mathrm{arc}\;\tanh\left(\frac{F_{crs}}{2}\sqrt{\frac{fr_{c}}{EIq\left(6\cos\alpha -5f\sin\alpha \right)}}\right)\right].$$ (57)

Over the interval $\left(z_{crs}^{0},z_{L}\right)$, the coiled tubing assumes a straight-line shape, and the axial load is

$$F(z)=\max\left\{0,F_{L}+(q\cos\alpha -fq\sin\alpha )(z_{L}-z)\right\}.$$ (58)

#### The third case of compressive force

When *F*_*crs*_ − (*q* cos *α* − *fq* sin *α*)(*z*_*L*_ − *z*_max_) ≤ *F*_*L*_ < *F*_*crs*_, only the part of the coiled tubing near the bottom end (0 < *z* ≤ *z*_*crh*,1_) takes on a helical shape, whereas the middle section (*z*_*crh*,1_ < *z* ≤ *z*_*crs*,1_) forms a sinusoidal shape. Near the loading end (*z*_*crh*,1_ < *z* ≤ *z*_*L*_), the coiled tubing exhibits a straight-line shape. The points *z*_*crs*,1_ and *z*_*crh*,1_ can be obtained by solving Eqs 59 and 60, respectively.

$$z_{crs,1}=z_{L}-\frac{F_{crs}-F_{L}}{q\cos\alpha -fq\sin\alpha },$$ (59)

$$F_{crh}=2\sqrt{\frac{EIq\left(6\cos\alpha -5f\sin\alpha \right)}{fr_{c}}}\tanh\left[\frac{z_{crs,1}—z_{crh,1}}{12}\sqrt{\frac{fr_{c}q\left(6\cos\alpha -5f\sin\alpha \right)}{EI}}+\mathrm{arc}\;\tanh\left(\frac{F_{crs}}{2}\sqrt{\frac{fr_{c}}{EIq\left(6\cos\alpha -5f\sin\alpha \right)}}\right)\right].$$ (60)

Similarly, the coiled tubing takes on a helical shape over the interval (0, *z*_*crh*,1_). Therefore, the axial load along this section of the coiled tubing is

$$F_{crh,1}\left(z\right)=2\sqrt{\frac{EIq\cos\alpha }{fr_{c}}}\tanh\left[\frac{z_{crh,1}-z}{2}\sqrt{\frac{fr_{c}q\cos\alpha }{EI}}+\mathrm{arc}\;\tanh\left(\frac{F_{crh}}{2}\sqrt{\frac{fr_{c}}{EIq\cos\alpha }}\right)\right].$$ (61)

Over the interval (*z*_*crh*,1_, *z*_*crs*,1_), the coiled tubing takes on a sinusoidal shape and the axial force is

$$F_{crs,1}(z)=2\sqrt{\frac{EIq\left(6\cos\alpha -5f\sin\alpha \right)}{fr_{c}}}\tanh\left[\frac{z_{crs,1}-z}{12}\sqrt{\frac{fr_{c}q\left(6\cos\alpha -5f\sin\alpha \right)}{EI}}+\mathrm{arc}\;\tanh\left(\frac{F_{crs}}{2}\sqrt{\frac{fr_{c}}{EIq\left(6\cos\alpha -5f\sin\alpha \right)}}\right)\right].$$ (62)

Over the interval (*z*_*crs*,1_, *z*_*L*_), the coiled tubing remains straight and the axial force is

$$F(z)=\max\left\{0,F_{L}+(q\cos\alpha -fq\sin\alpha )(z_{L}-z)\right\}.$$ (63)

#### The fourth case of compressive force

When *F*_*L*_ > *F*_*crs*_, the pipe takes on a helical shape near the bottom end (0 < *z* ≤ *z*_*crh*,2_). However, the remainder (*z*_*crh*,2_ < *z* ≤ *z*_*L*_) assumes a sinusoidal shape. The point *z*_*crh*,2_ can be calculated from

$$F_{crh}=2\sqrt{\frac{EIq\left(6\cos\alpha -5f\sin\alpha \right)}{fr_{c}}}\tanh\left[\frac{z_{L}-z_{crh,2}}{12}\sqrt{\frac{fr_{c}q\left(6\cos\alpha -5f\sin\alpha \right)}{EI}}+\mathrm{arc}\;\tanh\left(\frac{F_{L}}{2}\sqrt{\frac{fr_{c}}{EIq\left(6\cos\alpha -5f\sin\alpha \right)}}\right)\right].$$ (64)

Over the interval (0, *z*_*crh*,2_), the coiled tubing takes on a helical shape and the axial force is

$$F_{crh,2}\left(z\right)=2\sqrt{\frac{EIq\cos\alpha }{fr_{c}}}\tanh\left[\frac{z_{crh,2}-z}{2}\sqrt{\frac{fr_{c}q\cos\alpha }{EI}}+\mathrm{arc}\;\tanh\left(\frac{F_{crh}}{2}\sqrt{\frac{fr_{c}}{EIq\cos\alpha }}\right)\right].$$ (65)

Over the interval (*z*_*crh*,2_, *z*_*L*_), the coiled tubing is sinusoidal in shape and the axial force is

$$F_{crs,2}(z)=2\sqrt{\frac{EIq\left(6\cos\alpha -5f\sin\alpha \right)}{fr_{c}}}\tanh\left[\frac{z_{L}-z}{12}\sqrt{\frac{fr_{c}q\left(6\cos\alpha -5f\sin\alpha \right)}{EI}}+\mathrm{arc}\;\tanh\left(\frac{F_{L}}{2}\sqrt{\frac{fr_{c}}{EIq\left(6\cos\alpha -5f\sin\alpha \right)}}\right)\right].$$ (66)

#### The fifth case of compressive force

When *F*_*L*_ > *F*_*crh*_, the entirety of the coiled tubing takes on a helical shape. The axial load along the coiled tubing can be expressed as

$$F_{crh,3}\left(z\right)=2\sqrt{\frac{EIq\cos\alpha }{fr_{c}}}\tanh\left[\frac{z_{L}-z}{2}\sqrt{\frac{fr_{c}q\cos\alpha }{EI}}+\mathrm{arc}\;\tanh\left(\frac{F_{L}}{2}\sqrt{\frac{fr_{c}}{EIq\cos\alpha }}\right)\right].$$ (67)

In the case of *α* < arctan(1 / *f*), the total axial friction is

$$\Delta F=F(0)-F_{L}.$$ (68)

## Results and Discussion

### Effects of the boundary conditions and friction on the dimensionless critical load

For the pinned-end boundary conditions, the dimensionless axial load can be expressed as in Eq 25. When *m* < *m*_*crh*_, the number of helical turns *k* increases from *k*_*crh*_ to *k*_*crh*_ + 1, *k*_*crh*_ + 2, etc., as the dimensionless axial load decreases. The dimensionless axial load *m* corresponding to *k* > *k*_*crh*_ can be calculated by replacing *k* in Eq 25 with *k*_*crh*_ + 1, *k*_*crh*_ + 2, etc.

**Fig 3:**
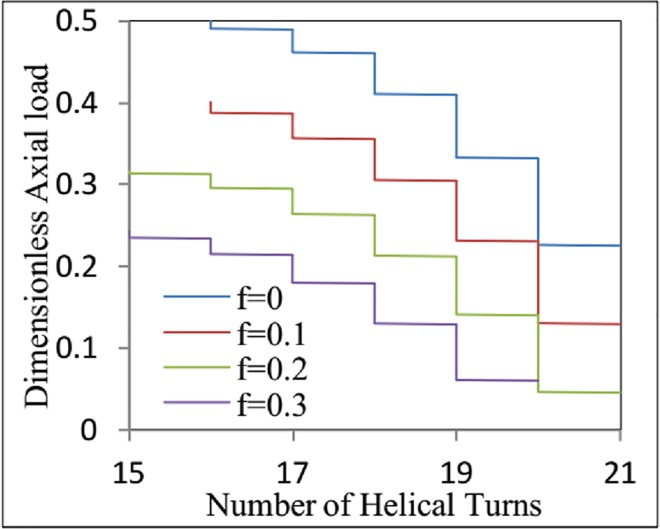
The relationship between the dimensionless axial force of the coiled tubing and the number of helical turns with the different friction coefficient.

Fig 3 shows the relationship between the dimensionless axial force on the coiled tubing and the number of helical turns. For a given dimensionless length of *ς*_*L*_ = 100, the dimensionless critical load is *m*_*crh*_ = 0.243 when *f*_2_ = 0.3. The critical number of the helical turns *k*_*crh*_ is 15. When *m* decreases to 0.234, the number of helical turns increases to 16, and when *m* further decreases to 0.214, the number of helical turns increases to 17. The corresponding relationships for the cases of *f*_2_ = 0, *f*_2_ = 0.1, and *f*_2_ = 0.2 are also clearly illustrated in Fig 3. For a given value of *m*, the number of helical turns *k* decreases as the friction coefficient increases.

**Fig 4:**
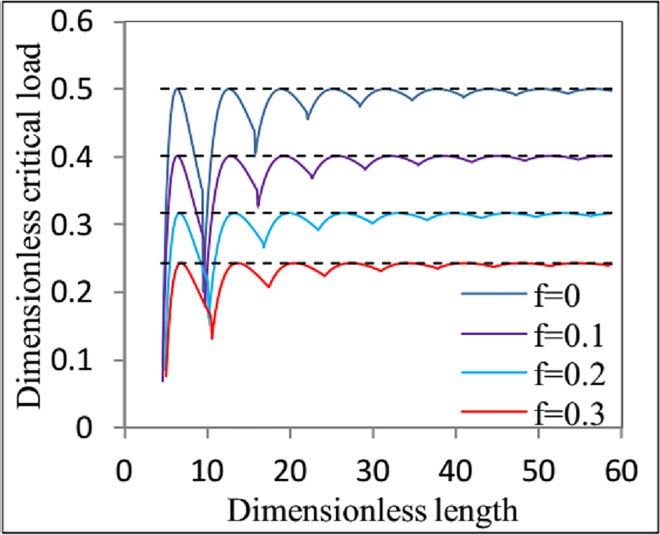
The relationship between the dimensionless critical load for helical buckling and the dimensionless length with the different friction coefficient.

As seen from Fig 4, the critical load for helical buckling is affected by the friction coefficient and the length of the coiled tubing. The dashed lines in Fig 4 represent the dimensionless critical loads for helical buckling in coiled tubing of infinite length (Eq 27). For a given friction coefficient, the dimensionless critical load approaches a stable value as *ς*_*L*_ → ∞. Therefore, for practical engineering applications, we can ignore the influence of the boundary conditions when *ς*_*L*_ > 40. When *ς*_*L*_ → ∞, the dimensionless critical load *m* decreases as the friction coefficient increases.

There is an inverse relationship between the dimensionless axial load *m* and the axial load *F*. In fact that, friction should increase the load from an intuitional perspective. The critical load is advanced with the growth of friction coefficient. If we want to prevent the buckling, we hope to increase the friction coefficient.

**Fig 5:**
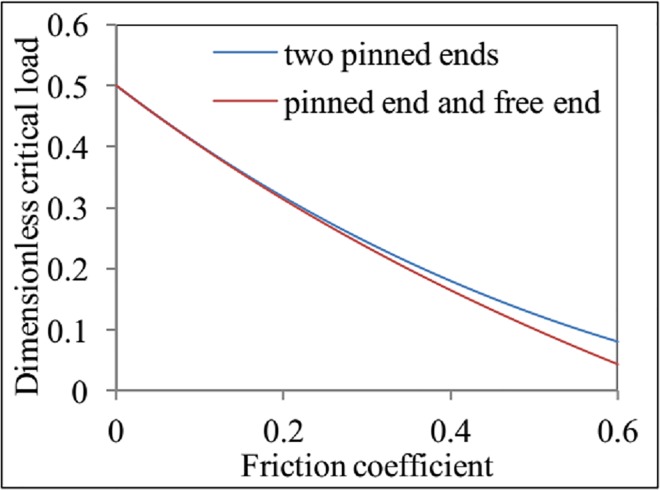
The effects of friction coefficient on the dimensionless critical load for helical buckling with different boundary conditions.

A comparison between the critical loads (Eqs 27 and 29) for two different sets of boundary conditions is shown in Fig 5. When *f*_2_ < 0.3, Eqs 27 and 29 give nearly the same critical load for helical buckling, whereas a difference appears between the results of Eqs 27 and 29 for *f*_2_ ≥ 0.3. This may be because the periodic terms were ignored during the calculation process (see Appendix E in S1 File). Another possible reason is that the boundary conditions for two pinned ends were applied to calculate the total energy.

### Effect of angular inclination on the critical load for helical buckling

In the case of the boundary conditions for two pinned ends, the critical load for helical buckling can be expressed using the terms $m=\frac{q\sin\alpha }{EIr_{c}\mu^{4}}$ and $\mu =\sqrt{\frac{F}{2EI}}$ in Eq 27.

$$F_{crh}=2\sqrt{\frac{q\sin\alpha EI\left[-36\pi f_{2}^{2}+\left(180-18\pi^{2}\right)f_{2}+90\pi \right]}{r_{c}\left(5\pi^{3}f_{2}^{2}-30\pi^{2}f_{2}+45\pi \right)}}.$$ (69)

**Fig 6:**
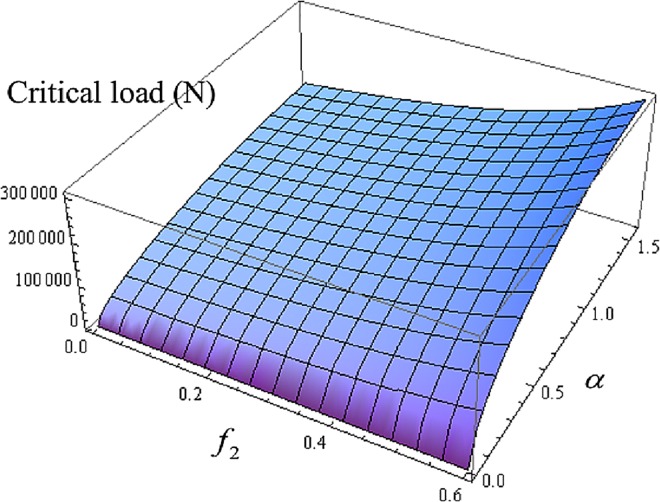
The combined effects of friction coefficient *f*_*2*_ and inclination angle *α* on the critical load for helical buckling *F*_*crh*_.

Here, an example of a 312—inch length of coiled tubing in a 634 inch inclined wellbore is considered. In this example, *E* = 2.1×10^11^ (N/m^2^), *I* = 1.81×10^−6^ (m^4^), *r*_*c*_ = 0.041272(m), *q* = 206.0(N/m), and $F_{crh}=87113.4\sqrt{\frac{\left[90\pi -36f_{2}^{2}\pi +f_{2}(180-18\pi^{2})\right]\sin(\alpha )}{45\pi -30f_{2}\pi^{2}+5f_{2}^{2}\pi^{3}}}$ (see Fig 6). Fig 6 shows the combined effect of the friction coefficient and the inclination angle on the critical load for helical buckling.

**Fig 7:**
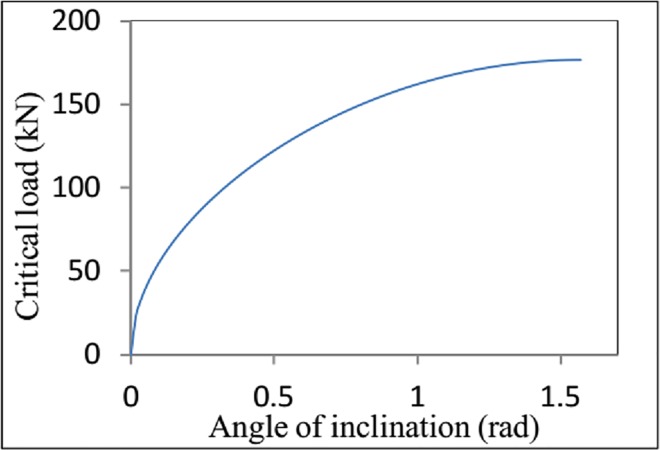
Variation in the critical load for helical buckling *F*_*crh*_ as a function of angle of inclination *α*.

**Fig 8:**
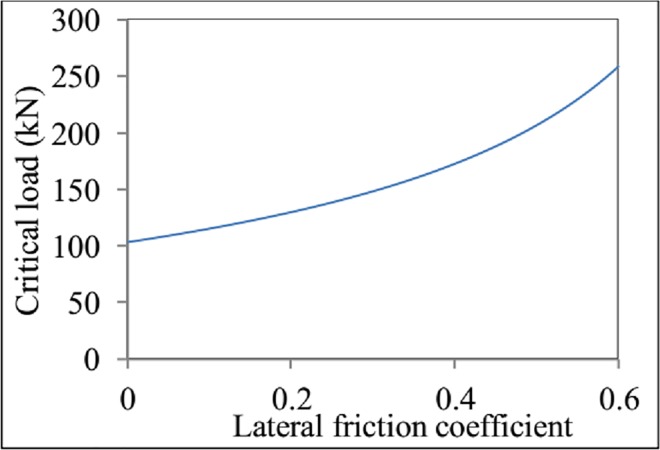
Variation in the critical load for helical buckling *F*_*crh*_ as a function of lateral friction coefficient *f*_*2*_.

For *f*_2_ = 0.3, the critical load for helical buckling is $F_{crh}=176593.7\sqrt{\sin\alpha }$. The relationship between the critical axial force (*F*_*crh*_) and the inclination angle (*α*) is shown in Fig 7. The critical load (*F*_*crh*_) is positively correlated with the angle of inclination (*α*), meaning that the critical load for helical buckling is affected by the contact force, because an increase in the angle of inclination causes the contact force to increase. For *α* = *π* / 4, the critical load for helical buckling is $F_{crh}=78415.8\sqrt{\left[5\pi +f_{2}(10-\pi^{2})-2f_{2}^{2}\pi \right]/\left(9-6f_{2}\pi +f_{2}^{2}\pi^{2}\right)}$. Similarly, the relationship between the critical load (*F*_*crh*_) and the lateral friction coefficient (*f*_2_) is shown in Fig 8. The critical load for helical buckling increases with an increasing the friction coefficient.

The buckling shape depends on the ratio of *r* / *R*. Thus, this ratio will impact on the critical load for helical buckling. The parameter *r*_*c*_ is the distance which is between the axis of the coiled tubing and the borehole axis. Therefore, we can discuss the impact of the distance *r*_*c*_ on the critical load for helical buckling. Eq 27 shows that the critical load will increase along with the decrease of the distance *r*_*c*_. If *R* is equal to *r*, then *r*_*c*_ is zero. The critical load for helical buckling will tend to infinity. The buckling will never happen. Therefore, we usually want to use the column diameter as large as possible in the project.

From the analysis above, it is easily seen that the friction coefficient, the distance *r*_*c*_, and the inclination angle are important factors. Therefore, we must consider these factors when we designed the well trajectory and downhole operation.

**Fig 9:**
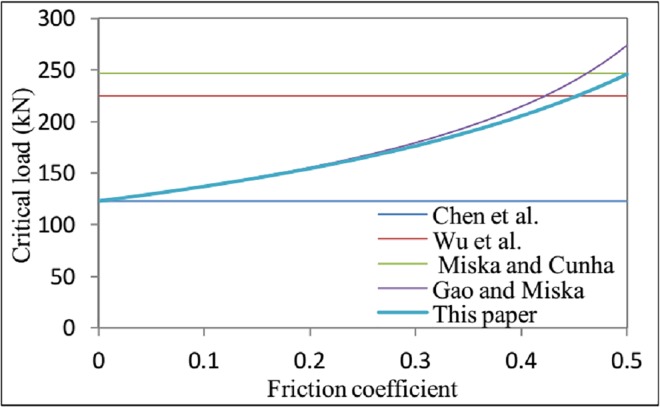
The effect of friction coefficient *f*_*2*_ on the critical load for helical buckling *F*_*crh*_.

When *α* = 90° and *f*_2_ = 0, the inclined wellbore degenerates into a horizontal wellbore, and Eq 69 becomes $F_{crh}=2\sqrt{2qEI/r_{c}}$. This result is the same as that obtained by Chen et al. Many different solutions for helical buckling under different conditions have been derived by many investigators [12, 23, 24,25]. Fig 9 clearly illustrates these differences. Gao and Miska [23], Miska and Cunha [12], Wu et al. [24], and Chen et al. [25] have performed detailed studies of the problem of the critical load for helical buckling. Their conclusions are, respectively,

$$F_{crh}=2\sqrt{\frac{30(\pi +2f)\;EIq}{\pi r_{c}(15-7\pi f)}},\;F_{crh}=4\sqrt{\frac{2EIq}{r_{c}}},\;F_{crh}=(8-2\sqrt{2})\sqrt{\frac{EIq}{r_{c}}},\;\mathrm{and}\;F_{crh}=2\sqrt{\frac{2EIq}{r_{c}}}.$$ (70)

The conclusions of Chen et al. (1990), Wu et al. (1993), and Miska and Cunha (1995) were derived without considering friction. Therefore, these critical load values are not affected by the friction coefficient, and the results appear as horizontal lines. By contrast, the effect of friction was considered by Gao et al. as well as in the present work. When *f*_2_ ≤ 0.5, the result obtained by Gao et al. is in good agreement with that of this paper. When *f*_2_ = 0, the results reported by Chen et al. and Gao et al. are both consistent with the result derived in this paper.

### Effect of the inclination angle on the axial load during helical buckling

The contact force between the coiled tubing and the wellbore is a constant when the coiled tubing remains straight. However, the contact force increases with increasing axial load in buckled coiled tubing. The analytical solutions for the axial load in the different post-buckling configurations are derived above. From the loading end to the dead end, the axial load slowly decreases as a result of friction when $\arctan\frac{1}{f}\leq \alpha$, whereas the axial load on the coiled tubing gradually increases when $\alpha <\arctan\frac{1}{f}$.

To investigate the effect of the inclination angle on the axial load during helical buckling, the axial load is analyzed for these two load cases ($\arctan\frac{1}{f}\leq \alpha$ and $\alpha <\arctan\frac{1}{f}$). As an example, a 312 inch coiled tubing in a 634 inch inclined wellbore is considered. The length of the coiled tubing is 1000 m. In this example, *E* = 2.1×10^11^ (N/m^2^), *I* = 1.81×10^−6^ (m^4^), *q* = 206.0(N/m), *r*_*c*_ = 0.041272(m), and the friction coefficient is *f* = 0.3. The force at the loading end is *F*_*L*_ = 85 k*N*. The inclination angle satisfies $\alpha <\arctan\frac{1}{f}$. The axial load on the helically buckled coiled tubing can be obtained in accordance with the above analysis.

**Fig 10:**
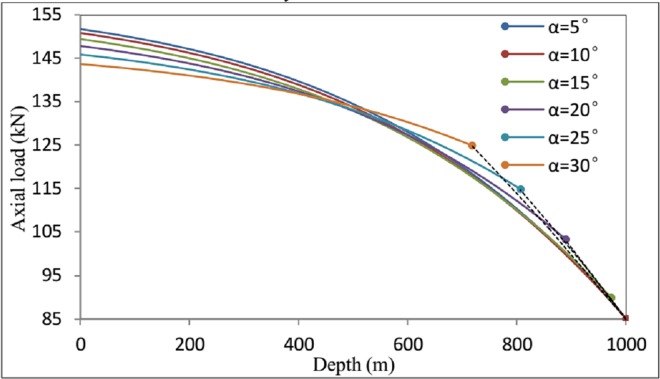
Variation of the axial load *F(z)* as functions of the depth *z* and the angular inclination *α*.

Fig 10 illustrates the distinction between the different axial load conditions. The solid lines in Fig 10 represent cases in which the shape of the coiled tubing becomes helical, whereas the dashed lines represent the axial force conditions under which the coiled tubing undergoes sinusoidal buckling or remains in an unbuckled state. The following conclusions can be drawn from an analysis of Fig 10. Firstly, as the angular inclination (*α*) increases, the critical location for helical buckling moves closer to the bottom of the wellbore. Secondly, as the angular inclination increases, the axial force increases nonlinearly from the loading end to the other end, with a continually decreasing growth rate. Finally, as the angular inclination increases, the axial force at the bottom is gradually reduced. Therefore, the total axial friction decreases as the angular inclination grows.

In summary, the angular inclination has a great impact on the helical buckling of coiled tubing. With the increase of angular inclination, the length of coiled tubing which is a helical shape is becoming increasingly shorter. The reason for this phenomenon is that the z axis component of gravitational force has changed. According to these findings, we could help better predict the helical buckling of coiled tubing. We can clearly understand the forces downhole string. This has important implications for the prevention of the fails of downhole operation.

## Conclusions

1. Equations for the buckling of coiled tubing under the influence of an axial load were developed in this work. The buckling behavior of the coiled tubing was illustrated by solving strongly nonlinear ordinary differential equations.
2. An analytical solution to the coiled tubing buckling equation was obtained for a helical post-buckling configuration using the perturbation method. Thus, a complete quantitative description of the helical buckling behavior of coiled tubing in an inclined wellbore was derived.
3. The effect of the boundary conditions on the helical buckling of coiled tubing is very small. For practical engineering applications, it can be ignored when the dimensionless length of the coiled tubing is greater than 40. Moreover, the influence of the boundary conditions on the dimensionless critical load can be ignored when *f*_2_ < 0.3. The effects of lateral friction and angular inclination on the critical load were obtained for a helical configuration by analyzing the critical load. The critical load for helical buckling increases with increasing lateral friction and with an increasing angle of inclination.
4. The axial force was studied for different inclination angles. It was determined that as the angle of inclination increases, the length of the coiled tubing that is in the helical buckling state decreases, the axial force varies gradually, and the total axial friction decreases.

## Supporting Information

S1 File This is the appendix A-E.
(DOCX)

## Abbreviations

α: lateral angular displacement
F_L_: axial compressive force at the top of the coiled tubing (N)
k: number of half-sinusoidal waves
L: length of the coiled tubing (m)
m: dimensionless axial compressive load
n: dimensionless normal contact force
f: friction coefficient
r_c_: clearance between the coiled tubing and the wellbore (m)
N: distributed normal contact force (N/m)
r_p_: inner radius of the coiled tubing (m)
q: effective weight per unit length of the coiled tubing (N/m)
*f*_1_: axial component of the friction coefficient
R_p_: outer radius of the coiled tubing (m)
ς: dimensionless length
u: axial displacement (m)
*f*_2_: lateral component of the friction coefficient
$\vec{\kappa }$: unit vector in the tangential direction
θ: angular displacement
