# Supplementary material for: The Effects of Boundary Conditions and Friction on the Helical Buckling of Coiled Tubing in an Inclined Wellbore
Source: PLoS One. 2016 Sep 20;11(9):e0162741. doi: 10.1371/journal.pone.0162741 (PMC5029948; doi:10.1371/journal.pone.0162741)
Supplement: S1 File — This is the appendix A-E. (DOCX) [file pone.0162741.s001.docx]

# Appendix A: Buckling Equations

The total energy of the pipe can be expressed as

$\prod=U-W$.

The elastic potential energy is

$U=U_{a}+U_{b}$.

Eq. A-3 describes the elastic compressive deformation energy of the coiled tubing. Its variation can be expressed as shown in Eq. A-4.

$U_{a}=\frac{1}{2}\int_{0}^{L} \left[F\left(z\right)\frac{du_{a}}{dz}\right]dz$,

.

The elastic compressive bending energy is

$\begin{matrix} U_{b}=\frac{EI}{2}\int_{0}^{L} k^{2}dz=\frac{EI}{2}\int_{0}^{L} \left(k_{r}^{2}+k_{\theta}^{2}\right)dz=\frac{EI}{2}\int_{0}^{L} r^{2}\left[\left(\frac{d^{2}\theta}{dz^{2}}\right)^{2}+\left(\frac{d\theta}{dz}\right)^{4}\right]dz \\ +EI\int_{0}^{L} r\left[2\frac{dr}{dz}\frac{d\theta}{dz}\frac{d^{2}\theta}{dz^{2}}-\left(\frac{d\theta}{dz}\right)^{2}\frac{d^{2}r}{dz^{2}}\right]dz+\frac{EI}{2}\int_{0}^{L} \left[\left(\frac{d^{2}r}{dz^{2}}\right)^{2}+4\left(\frac{d\theta}{dz}\right)^{2}\left(\frac{dr}{dz}\right)^{2}\right]dz \end{matrix}$,

where$EI=\frac{E\pi}{4}\left(R_{P}^{4}-r_{P}^{4}\right)$ is the bending stiffness in $\text{N.} \text{m}^{2}$; here, $r_{p}$ is the inner radius of the coiled tubing, in $\text{m}$, and $R_{p}$ is the outer radius, also in $\text{m}$.

,

,

.

The total variation of $U$ is given by

$\begin{matrix} \delta U=\delta U_{a}+\delta U_{b}=\int_{0}^{L} \frac{dF\left(z\right)}{dz}\delta U_{a}\left(z\right)dz+4EI\int_{0}^{L} \left\{\frac{d^{3}r}{dz^{3}}\frac{d\theta}{dz}+\frac{3}{2}\frac{d^{2}r}{dz^{2}}\frac{d^{2}\theta}{dz^{2}}\right\}r\delta\theta dz+4EI\int_{0}^{L} \left\{\frac{dr}{dz}\left[\frac{d^{3}\theta}{dz^{3}}-\left(\frac{d\theta}{dz}\right)^{3}\right]\right\}r\delta\theta dz \\ +EI\int_{0}^{L} \left\{\frac{d^{4}\theta}{dz^{4}}-2\frac{d}{dz}\left[\left(\frac{d\theta}{dz}\right)^{3}\right]\right\}r^{2}\delta\theta dz+EI\int_{0}^{L} \left\{\frac{d^{4}r}{dz^{4}}-6\frac{d}{dz}\left[\frac{dr}{dz}\left(\frac{d\theta}{dz}\right)^{2}\right]\right\}\delta rdz+EI\int_{0}^{L} \left[\left(\frac{d\theta}{dz}\right)^{4}-3\left(\frac{d^{2}\theta}{dz^{2}}\right)^{2}-4\frac{d\theta}{dz}\frac{d^{3}\theta}{dz^{3}}\right]r\delta rdz \end{matrix}$.

**The work done by the force** **in the X direction**

Let $W_{G_{2}}$ represent the work done by gravity in the X direction; its variation is expressed as shown in Eq. A-10. The work done by the normal contact force $W_{N}$ and its variation is given by Eq. A-11.

$W_{G_{2}}=-\int_{0}^{L} q\sin\alpha\left(r_{c}-r\cos\theta\right)dz, \delta W_{G_{2}}=q\sin\alpha\int_{0}^{L} \cos\theta\delta rdz-q\sin\alpha\int_{0}^{L} r\sin\theta\delta\theta dz$.

.

**The work done by the force** **in the Y direction**

Let $W_{x,f_{2}}$ represent the work done by the lateral friction force. Its variation is given by Eq. A-13.

.

.

**The work done by the force** **in the Z direction**

For a given axial displacement profile $u_{a}(z)$, the work done by the force along the Z direction is denoted by $W_{Fz,a}$. Similarly, for the displacements defined by $u_{b}(z)$, $W_{Fz,b}$ denotes the work. These quantities and their variations are expressed as follows:

$W_{Fz}=W_{Fz,a}+W_{Fz,b}$,

,

,

where $F_{L}$ represents the axial force at the loading end, and

$W_{Fz,b}=\int_{0}^{u_{b}(L)} F_{L}du_{b}(L)-\int_{0}^{L} \int_{0}^{u_{b}(z)} f_{1}\left(z\right)N\left(z\right)du_{a}(z)dz+\int_{0}^{L} \int_{0}^{u_{b}(z)} q\cos\alpha du_{a}(z)dz$,

.

By interchanging the order of integration, this expression can be rewritten as follows:

$\int_{0}^{L} H(x)\int_{0}^{x} G\left(s\right)dsdx=\int_{0}^{L} \int_{0}^{x} H(x)G\left(s\right)dsdx=\int_{0}^{L} \int_{s}^{L} H(x)G\left(s\right)dxds=\int_{0}^{L} G\left(s\right)\int_{s}^{L} H(x)dxds$,

.

The axial force is defined as

$F\left(z\right)=F_{L}-\int_{z}^{L} \left(f_{1}(z)N(z)-q\cos\alpha\right)dz$.

Let $u_{b}(z)$ denote the axial displacement induced by buckling or lateral bending.

$u_{b}(z)=\frac{1}{2}\int_{0}^{z} \left[\left(\frac{dr}{dz}\right)^{2}+r^{2}\left(z\right)\left(\frac{d\theta}{dz}\right)^{2}\right]dz, \frac{du_{b}(z)}{dx}=\frac{1}{2}\left[\left(\frac{dr}{dz}\right)^{2}+r^{2}\left(z\right)\left(\frac{d\theta}{dz}\right)^{2}\right]$,

,

.

The total variation of is

$\delta\prod=\delta U-\delta W$.

$\delta u_{a}(z)$, $\delta r$, and $\delta\theta$ can respectively be expressed as

$\int_{0}^{L} \frac{dF\left(z\right)}{dz}\delta u_{a}\left(z\right)dz-\int_{0}^{L} f_{1}\left(z\right)N\left(z\right)\delta u_{a}(z)dz+\int_{0}^{L} q\cos\alpha\delta u_{a}(z)dz=0$,

,

.

The buckling equations can thus be obtained using the principle of virtual work.

$\frac{dF\left(z\right)}{dz}=f_{1}\left(z\right)N\left(z\right)-q\cos\alpha$,

,

.

# Appendix B: Work and Elastic Deformation Energy

We assume that the coiled tubing is in continuous contact with the wall. In other words, $r$ is a constant ($r=r_{c}$). Therefore, the elastic deformation energy and its variation are, respectively,

$U_{b}=\frac{EI}{2}\int_{0}^{L} k^{2}dz=\frac{EI}{2}\int_{0}^{L} \left(k_{r}^{2}+k_{\theta}^{2}\right)dz=\frac{EI}{2}\int_{0}^{L} r^{2}\left[\left(\frac{d^{2}\theta}{dz^{2}}\right)^{2}+\left(\frac{d\theta}{dz}\right)^{4}\right]dz$,

.

Similarly, the work done by the axial load and its variation can be expressed as

$W_{Fz,b}=\int_{0}^{u_{b}(L)} F_{L}du_{b}(L)-\int_{0}^{L} \int_{0}^{u_{b}(z)} f_{1}\left(z\right)N\left(z\right)du_{a}(z)dz+\int_{0}^{L} \int_{0}^{u_{b}(z)} q\cos\alpha du_{a}(z)dz$,

.

# Appendix C: Derivation of the Potential Energy

When the effect of the lateral friction is considered, the total dimensionless potential energy is defined as

$\Omega=\frac{1}{2m\varsigma_{L}}\int_{0}^{\varsigma_{L}} \left[\left(\frac{d^{2}\theta}{d\varsigma^{2}}\right)^{2}+\left(\frac{d\theta}{d\varsigma}\right)^{4}-2\left(\frac{d\theta}{d\varsigma}\right)^{2}\right]d\varsigma+\frac{1}{m\varsigma_{L}}\int_{0}^{\varsigma_{L}} sign\left(\theta\right)\int_{0}^{\theta\left(\varsigma\right)} f_{2}nd\vartheta d\varsigma+\frac{1}{\varsigma_{L}}\int_{0}^{\varsigma_{L}} \left(1-\cos\theta\right)d\varsigma$,

where $n(\vartheta)$ represents the dimensionless normal contact force on the coiled tubing, and

$n=3\left(\frac{d^{2}\vartheta}{d\varsigma^{2}}\right)^{2}+4\frac{d\vartheta}{d\varsigma}\frac{d^{3}\vartheta}{d\varsigma^{3}}-\left(\frac{d\vartheta}{d\varsigma}\right)^{4}+m\cos\vartheta+2\left(\frac{d\vartheta}{d\varsigma}\right)^{2}$.

When the coiled tubing enters a state of helical buckling, it can be assumed that the configuration of the pipe will take the following form:

$\vartheta(\varsigma,t)=p(t)\varsigma$,

where $\left|p\right|$ will range from 0 to $\left|p_{h}\right|$. By substituting Eq. C-3 into Eq. C-2, we obtain

$n=2p^{2}-p^{4}+m\cos\vartheta$.

For a given $\varsigma$, $d\vartheta=\varsigma dp$, and

$sign(\theta)\int_{0}^{\theta_{(\varsigma)}} nd\vartheta=\int_{0}^{\left|\theta_{(\varsigma)}\right|} nd\vartheta=\int_{0}^{\left|p_{h}\right|} (2p^{2}-p^{4})\varsigma dp+m\sin\left|p_{h}\xi\right|=\left(\frac{2}{3}p_{h}^{2}-\frac{1}{5}p_{h}^{4}\right)\left|p_{h}\right|\varsigma+m\sin\left|p_{h}\xi\right|$.

The integer $k$ represents the number of helical turns of the coiled tubing with a given length $\varsigma_{L}$:

$\varsigma_{L}=\frac{2k\pi}{\left|p_{h}\right|}=2k\varsigma_{h}$,

where $\varsigma_{h}=\frac{\pi}{p_{h}}$ denotes the length of one half -helix turn.

$\int_{0}^{\varsigma_{L}} sign\left(\theta\right)\int_{0}^{\theta\left(\varsigma\right)} nd\vartheta d\varsigma=2k\int_{0}^{\varsigma_{h}} \int_{0}^{\left|\theta\left(\varsigma\right)\right|} nd\vartheta d\varsigma=k\left(\frac{2}{3}p_{h}^{2}-\frac{1}{5}p_{h}^{4}\right)\left|p_{h}\right|\varsigma_{h}^{2}+\frac{4k}{\left|p_{h}\right|}=\frac{\pi}{2}\left(\frac{2}{3}p_{h}^{2}-\frac{1}{5}p_{h}^{4}\right)\varsigma_{L}+m\frac{2\varsigma_{L}}{\pi}$,

,

.

Thus, we arrive at the total dimensionless potential energy for the helical buckling of the coiled tubing by substituting Eqs. C-6 to C-9 into Eq. C-1.

$\Omega_{h}=\left(\frac{1}{2m}-\frac{\pi f_{2}}{10m}\right)p_{h}^{4}+\left(\frac{\pi f_{2}}{3m}-\frac{1}{m}\right)p_{h}^{2}+\frac{2f_{2}}{\pi}+1$.

# Appendix D: Solution for Sinusoidal Buckling

If buckling occurs, axial friction becomes dominant. Therefore, for post-buckling analysis, we can assume that the lateral friction can be neglected,$f_{2}=0$, and $f_{1}\approx f$ . Then, Eq. 8 can be simplified to

$\frac{d^{4}\theta}{d\varsigma^{4}}-6\left(\frac{d\theta}{d\varsigma}\right)^{2}\frac{d^{2}\theta}{d\varsigma^{2}}+2\frac{d^{2}\theta}{d\varsigma^{2}}+m\sin\theta=0$.

Once buckling is induced, the wavelength of the sine wave will remain unchanged. However, the amplitude will increase with increasing axial load. Therefore, during the initiation of sinusoidal buckling, the solution to Eq. D-1 can be assumed to have the following form:

$\theta\left(\varsigma,t\right)=a\left(t\right)\sin\left(\varsigma\right)+a^{2}\left(t\right)g_{2}\left(\varsigma,t\right)+a^{3}\left(t\right)g_{3}\left(\varsigma,t\right)+o\left(a^{4}\right)$.

Substituting Eq. D-2 into Eq. D-1 yields

$\begin{matrix} a\left[m-1+\frac{3a^{2}}{2}\right]\sin\left(\varsigma\right)+a^{2}\left[\frac{d^{4}g_{2}\left(\varsigma,t\right)}{d\varsigma^{4}}+2\frac{d^{2}g_{2}\left(\varsigma,t\right)}{d\varsigma^{2}}+mg_{2}\left(\varsigma,t\right)\right] \\ +a^{3}\left[\frac{d^{4}g_{3}\left(\varsigma,t\right)}{d\varsigma^{4}}+2\frac{d^{2}g_{3}\left(\varsigma,t\right)}{d\varsigma^{2}}+mg_{3}\left(\varsigma,t\right)+\frac{3}{2}\sin(3\varsigma)\right]+o(a^{4})=0 \end{matrix}$.

The solution to Eq. D-3 can be expressed as follows:

$a=\sqrt{\frac{2\left(1-m\right)}{3}}, g_{2}\left(\varsigma,t\right)=0, g_{3}\left(\varsigma,t\right)=\frac{(126+2m)a\sin\varsigma+(1-m)3\sin(3\varsigma)}{2m^{2}+124m-126}$.

Because the term $a^{3}(t)g_{3}(\varsigma,t)$ is on the order of $10^{-3}$, it can be ignored in practical engineering applications. Therefore, the solution to Eq. D-1 can be approximated as

$\theta(\varsigma,t)\text{=}\sqrt{\frac{2\left(1-m\right)}{3}}\sin(\varsigma)$.

# Appendix E: Solution for Helical Buckling

The coiled tubing will transition from a sinusoidal shape to a helical buckling shape when the axial force exceeds the critical load for helical buckling.

$\frac{d^{4}\theta}{d\varsigma^{4}}-6\left(\frac{d\theta}{d\varsigma}\right)^{2}\frac{d^{2}\theta}{d\varsigma^{2}}+2\frac{d^{2}\theta}{d\varsigma^{2}}+m\sin\theta=0$.

Because buckling occurs for $m<1$, the solution to Eq. E-1 can be assumed to be of the form

$\theta(\varsigma)=\theta_{0}(\varsigma)+m\theta_{1}(\varsigma)+o(m^{2})$,

.

Substituting Eqs. E-2 and E-3 into Eq. E-1 results in

$\frac{d^{4}\theta_{0}}{d\varsigma^{4}}-6\left(\frac{d\theta_{0}}{d\varsigma}\right)^{2}\frac{d^{2}\theta_{0}}{d\varsigma^{2}}+2\frac{d^{2}\theta_{0}}{d\varsigma^{2}}=0$,

.

If we can solve Eq. E-4 for $\theta_{0}$, then subsequently solving Eq. E-5 for $\theta_{1}$ will be simple. Thus, let us now focus on seeking a solution to Eq. E-4 for $\theta_{0}$ for various boundary conditions. Integrating Eq. E-4 results in

$\frac{d^{3}\theta_{0}}{d\varsigma^{3}}+2\left[1-\left(\frac{d\theta_{0}}{d\varsigma}\right)^{2}\right]\frac{d\theta_{0}}{d\varsigma}-\rho_{1}=0, \rho_{1}=\left[\frac{d^{3}\theta_{0}}{d\varsigma^{3}}+2\left[1-\left(\frac{d\theta_{0}}{d\varsigma}\right)^{2}\right]\frac{d\theta_{0}}{d\varsigma}\right]_{\varsigma=0}$.

For the free end at $\varsigma=0$, under the natural boundary conditions (Eq. 19), we arrive at $\rho_{1}=0$. Otherwise, $\rho_{1}\neq0$. For $\eta=\frac{d\theta_{0}}{d\varsigma}$, $\frac{d^{3}\theta_{0}}{d\varsigma^{3}}=\frac{d}{d\varsigma}\left(\frac{d\eta}{d\varsigma}\right)=\frac{1}{2}\frac{d}{d\eta}\left[\left(\frac{d\eta}{d\varsigma}\right)^{2}\right]$ can be obtained by applying the chain rule. Therefore, Eq. E-6 can be rewritten as

$\frac{1}{2}\frac{d}{d\eta}\left[\left(\frac{d\eta}{d\varsigma}\right)^{2}\right]+2\eta-2\eta^{3}-\rho_{1}=0$,

,

.

For simplicity, this article discusses only the case of a length of coiled tubing with a free end at $\varsigma=0$. Therefore, the value of $\rho_{1}$ is zero. Fig E-1 presents the phase diagrams of Eq. E-8 for various constant values of $\rho_{2}$. The black-colored curves represent the case of $\rho_{2}\leq0$.

**Fig E-1. Phase diagram of** $\eta' \text{vs. }\eta$

The red curve in Fig E-1 , corresponding to $0<\rho_{2}<1$, assumes the shape of a closed loop. This means that Eq. E-4 has a periodic solution. This is not the main result of this analysis and corresponds to the sinusoidal buckling considered in our previous study. For $\rho_{2}>1$ (the green curves), there exists no value of $\eta$ for which $\eta'=0$ can be obtained. This indicates that there is no solution that satisfies the boundary conditions for a pinned or free end. For $\rho_{2}=1$ (the blue curve), $\eta'=\frac{d^{2}\theta_{0}}{d\varsigma^{2}}=0$ when $\eta=\pm1$. In other words, $\eta=\pm1$ is a specific solution that satisfies the boundary conditions for a pinned or free end. Because one end of the coiled tubing is free $\left(\rho_{1}=0\right)$, the helical buckling configuration of the massless coiled tubing can be expressed as

$\theta=\varsigma+c_{0}$.

For coiled tubing with two free ends, $c_{0}$ is an arbitrary constant. However, $c_{0}=0$ when one end is a pinned end. The expression $\rho_{1}\neq0$ corresponds to the other types of boundary conditions at $\varsigma=0$. However, for two pinned ends, the boundary conditions are a special case of $\rho_{1}\neq0$. For any integer $k$, the solution $\theta=\frac{2k\pi}{\varsigma_{L}}\varsigma$ is suitable not only for the two-pinned-end boundary conditions but also for the buckling equation given by Eq. E-4. Given an integer $k$, $\theta(\varsigma_{L})=2k\pi$ indicates that the end at $\varsigma=\varsigma_{L}$ will not undergo horizontal displacement. Substituting $\theta_{0}=\eta\varsigma+c_{0}$ into Eq. E-5 yields

$\frac{d^{4}\theta_{1}}{d\varsigma^{4}}+2\left(1-3\eta^{2}\right)\frac{d^{2}\theta_{1}}{d\varsigma^{2}}+\sin\left(\eta\varsigma+c_{0}\right)=0$.

The solution to Eq. E-11 is given by

$\theta_{1}=-\frac{\sin(\eta\varsigma+c_{0})}{\eta^{2}(7\eta^{2}-2)}$.

For the boundary conditions for pinned or free ends, the solution is

$$\theta(\varsigma)=\eta\varsigma+c_{0}-m\frac{\sin(\eta\varsigma+c_{0})}{\eta^{2}(7\eta^{2}-2)}$$

Compared with the linear term, the perturbation term is very small. Therefore, we can ignore this term for practical engineering applications.
